# Expression of Programmed Cell Death Protein 1 by Tumor-Infiltrating Lymphocytes and Tumor Cells is Associated with Advanced Tumor Stage in Patients with Esophageal Adenocarcinoma

**DOI:** 10.1245/s10434-017-5858-7

**Published:** 2017-04-20

**Authors:** Dagmar Kollmann, Desislava Ignatova, Julia Jedamzik, Yun-Tsan Chang, Gerd Jomrich, Matthias Paireder, Ivan Kristo, Dmitry Kazakov, Michal Michal, Antonio Cozzio, Wolfram Hoetzenecker, Tobias Schatton, Reza Asari, Matthias Preusser, Emmanuella Guenova, Sebastian F. Schoppmann

**Affiliations:** 1 Department of Surgery, Comprehensive Cancer Center Vienna, Upper-GI-Service, GET-Unit, Medical University of Vienna, Vienna, Austria; 2 Department of Dermatology, University Hospital Zürich, University of Zurich, Zurich, Switzerland; 3 Department of Pathology, Faculty of Medicine in Plzen and Charles University Hospital Plzen, Biomedical Center, Charles University, Pilsen, Czech Republic; 4 Department of Dermatology, Kantonspital St. Gallen, University of Zurich, Zurich, Switzerland; 5 Department of Dermatology, Kepler University Hospital, Linz, Austria; 6 Department of Dermatology, Harvard Skin Disease Research Center, Brigham and Women’s Hospital, Harvard Medical School, Boston, MA USA; 7 Department of Medicine, Boston Children’s Hospital, Harvard Medical School, Boston, MA USA; 8 Clinical Division of Oncology, Department of Medicine I and Comprehensive Cancer Center, GET-Unit, Medical University of Vienna, Vienna, Austria

## Abstract

**Background:**

Despite recent advances in the therapy for adenocarcinoma of the esophagogastric junction (AEG), overall prognosis remains poor. Programmed cell death protein 1 (PD1) is a co-inhibitory receptor primarily expressed by T-cells. Tumor cells can escape anticancer immune responses by triggering the PD1 pathway. Moreover, PD1 receptor engagement on cancer cells may trigger tumor-intrinsic growth signals. This study aimed to evaluate the potential clinical relevance of PD1 expression by tumor-infiltrating lymphocytes (TILs) and cancer cells in the AEG.

**Methods:**

Patients with AEG who underwent esophagectomy from 1992 to 2011 were included in the study. Expression of PD1was evaluated by immunohistochemistry and correlated with long-term overall survival (OS), disease-free survival (DFS), and various clinicopathologic parameters.

**Results:**

Tumor biospecimens from 168 patients were analyzed. In the analysis, 81% of the patients showed high tumoral frequencies (>5%) of PD1-expressing TILs (TIL-PD1^+^), and 77% of patient tumors harbored high levels (>5%) of PD1^+^ cancer cells (cancer-PD1^+^). Expression of PD1 by TILs and cancer cells correlated significantly (*p* < 0.05) with patients’ tumor stage and lymph node involvement. Compared with the patients who had low tumoral frequencies of PD1^+^ TILs or cancer cells, the TIL-PD1^+^ and cancer-PD1^+^ patients demonstrated significantly reduced DFS in the univariate analysis (5-year DFS: 73.3 vs. 41.9%, log-rank 0.008 and 71.3 vs. 41.6%, *p* = 0.008, respectively). Additionally, the cancer-PD1^+^ patients showed significantly decreased OS in the univariate analysis compared with the cancer-PD1^−^ patients (5-year OS: 68.8 vs. 43.5%; *p* = 0.047). However, these correlations did not reach significance in the multivariate analysis.

**Conclusions:**

The PD1 receptor is expressed by both TILs and cancer cells in AEG. High expression of PD1 is associated with advanced tumor stage and lymph node involvement, but not with survival.

Esophageal cancer is the eighth most common cancer worldwide, with dramatically increasing prevalence, and the sixth most common cause of cancer-related death.^1^ Besides surgical resection, therapeutic strategies include radiotherapy, cytotoxic chemotherapy,^2^ and treatment with the monoclonal antibody, trastuzumab, targeting human epidermal growth factor receptor 2 (HER2), which is overexpressed in about 1 5% of esophageal carcinomas.^3^ Despite these current therapies, overall survival (OS) rates remain low, with 5-year survival percentages ranging from approximately 10 to 15%.^2^ Accordingly, new molecular targets are urgently needed to improve patient outcome.

Recently, the programmed cell death 1 (PD1) protein, an immune checkpoint receptor primarily expressed by activated T-cells, has been described as a key mediator of tumor immune evasion and cancer progression.^4^,^5^ Expression of the PD1 ligands PD-L1 and PD-L2 by tumor-infiltrating lymphocytes (TILs) and cancer cells has been described for various tumor entities including melanoma, multiple myeloma, breast cancer, renal cell carcinoma, colorectal cancer, and lung carcinoma.^6^ High expression of PD1 ligands in the tumor microenvironment (TME) has been associated with adverse clinical outcome 7,8 and escape from tumor-specific T cell immunity via a process termed adaptive immune resistance.^9^,^10^ Therapeutic PD1-blocking antibodies have been developed to counteract this prominent tumor immune escape mechanism. In clinical trials, PD1 inhibitors have shown remarkable efficacy in patients with advanced cancers of various etiologies including melanoma, renal cell carcinoma, non-small cell lung cancer, and colorectal carcinoma.^11^,^12^

In esophageal carcinoma, data regarding PD1 expression in the TME is sparse, and little is known about the potential significance of PD1 as a cancer therapeutic target and mechanism of disease progression. A recent study by Chen et al.^13^ showed PD1 surface protein expression by TILs in 33.5% and PD-L1 expression by TILs and/or cancer cells in 41.4% of patients with esophageal squamous cell carcinoma. Tumoral PD-L1 expression levels correlated with postoperative outcome, but no significant associations between PD1 expression and clinicopathologic parameters or postoperative outcome were determined in this patient cohort. In contrast, a separate study involving gastric cancer patients showed that PD1 expression by CD4^+^ and CD8^+^ TILs correlated positively with tumor progression.^14^

In addition to the well-established role of PD1 in dampening T-cell responses,^10^,^15^ recent evidence suggests that the PD1 receptor also is expressed by cancer cell subsets^16^ and that tumor cell-intrinsic PD1 pathway activation promotes cancer progression by triggering tumor cell-intrinsic growth pathways, including PI3K/AKT/mTOR.^16^–^18^

This study aimed to characterize PD1 expression profiles in tumor biospecimens obtained from patients with resectable adenocarcinoma of the esophagogastric junction (AEG) and to assess whether TIL and/or tumoral PD1 expression levels correlate with patient prognosis, including disease-free survival, OS, and other clinicopathologic parameters.

## Methods

### Study Population

Consecutive patients from a prospective database who had received esophageal resection for AEG between 1992 and 2011 in the Department of Surgery at the Medical University of Vienna were included in this analysis. Finally, 168 patients were identified, and data were collected using the institutional database and review of individual patients’ charts. The follow-up evaluation was performed according to the institutional policy. All patients were followed at 3-months interval during the first year after esophagectomy, at 6-month intervals during the next 3 years after esophagectomy, and yearly thereafter. Tissue samples of resected tumors were collected and used for histologic analysis. The study was approved by the local ethics committee of the Medical University of Vienna (#1056/2016).

### Immunohistochemistry (IHC)

For immunohistochemical analyses, 3- to 5-μm-thick paraffin sections were used as previously described.^19^ Expression of PD1 was detected by using anti-human PD1 antibody (R&D Systems, #AF 1086, dilution 1:20). Antigen retrieval was achieved by heating slides in a Dako Cytomation Pascal Pressure Cooker, and 3% hydrogen peroxide in distilled water was used to block the endogenous peroxidase activity. Nonspecific epitopes were blocked with normal goat serum (30 min), and the sections then were incubated successively with primary antibody (1:20 dilution, 60 min, room temperature) and corresponding biotinylated anti-goat immunoglobulin G secondary antibody (1:100 dilution, 30 min). According to the manufacturer’s protocol (Dako), visualization via streptavidin conjugated to alkaline phosphatase was implemented. To depict the cell nuclei, additional Mayer’s hematoxylin staining was applied.

For each slide, four different areas of esophageal adenocarcinoma were selected for analysis. The immunoreactivity for PD1 of tumor cells and lymphocytes was examined at ×400 magnification, and the staining rate (percentage of tumor cells and lymphocytes showing positive staining, 0–100%) was determined. Expression of PD1 was categorized as 0 (no positive cells), 1 + (5–25% of cells), 2 + (26–50% of cells), 3 + (51–75% of cells), and 4 + (76–100% of cells). Three pathologists blinded to the clinical characteristics of each patient evaluated the staining for both tumor cells and lymphocytes and independently graded each slide. If the rating differed, the slide was re-discussed using a multi-head microscope, and a consensus was found. The cutoff for positive PD1 expression was set at 5% based on the expertise of the pathologists, which showed 5% as the cutoff to be practical with the staining patterns, especially in terms of a future clinical application. Additionally, previous publications on PD1-L expression as a biomarker for treatment have identified a cutoff of 5%.^20^–^22^

### Statistical Analysis

Statistical analysis was performed with SPSS 20 (SPSS, Inc., Chicago IL, USA). The association of PD1 expression and clinicopathologic parameters was analyzed using the Chi square test, *t* test, or Mann–Whitney *U* test. For the calculation of OS, the time between primary surgery and the patient’s death was analyzed. Disease-free survival (DFS) was defined as the time from primary surgery until the first evidence of disease progression. For the calculation of both OS and DFS, patients without complete resection (*n* = 23) were excluded from the analyses. The influence of PD1 expression on tumor cells and lymphocytes as well as the influence of other clinicopathologic findings on OS and DFS was evaluated with the Kaplan–Meier method, log-rank tests, and the Cox proportional hazard model. All tests were performed in a two-sided manner, and *p* values lower than 0.05 were considered to be statistically significant.

## Results

### Patients’ Characteristics

The study included 168 patients with esophageal adenocarcinoma. The ratio of female to male patients was 31:137, and the mean age at surgery was 65 ± 10.4 years (range 35–88 years). The median follow-up time was 29.4 months (range 0–196 months). The patients with complete resection (*n* = 145) had a 1-year OS rate of 78.6% (107 of 145 patients at risk) a 5-year OS rate of 49.9% (54 of 145 patients at risk), and a 10- year OS rate of 37.3% (12 of 145 patients at risk). These patients had 1-year DFS rate of 72.6% (79 of 145 patients at risk), a 5-year DFS rate of 48.7% (31 of 145 patients at risk), and a 10-year DFS rate of 39.7% (6 of 145 patients at risk). Of the 168 patients, 63 (37.5%) had received neoadjuvant therapy (59 had neoadjuvant chemotherapy and 4 had neoadjuvant radiochemotherapy). The clinical and histopathologic data are summarized in Tables 1 and 2.

**Table 1:** Association of programmed cell death protein 1 (PD1) expression by tumor infiltrating lymphocytes (TILs) with clinicopathologic parameters in 182 patients with esophageal adenocarcinoma

Factor	Adenocarcinoma (*n* = 168) *n* (%)	PD1^+^ TILs (*n* = 136) *n* (%)	PD1^−^ TILs (*n* = 32) *n* (%)	*p* value
Tumor stage				
High-grade dysplasia	4 (2.4)	1 (25)	3 (75)	<0.001
pT1a	13 (7.7)	5 (38.5)	8 (61.5)	
pT1b	20 (11.9)	7 (35)	13 (65)	
pT2	49 (29.2)	45 (91.8)	4 (8.2)	
pT3	77 (45.8)	76 (98.7)	1 (1.3)	
pT4	5 (3)	2 (40)	3 (60)	
Lymph node status				
pNx	13 (7.7)			0.007
pN0	61 (36.3)	41 (67.2)	20 (32.8)	
pN1	31 (18.5)	28 (90.3)	3 (9.7)	
pN2	26 (15.5)	24 (92.3)	2 (7.7)	
pN3	37 (22)	32 (86.5)	5 (13.5)	
Histologic grading				
G1	7 (4.2)	5 (71.4)	2 (28.6)	0.350
G2	74 (44)	57 (77)	17 (23)	
G3	87 (51.8)	74 (85.1)	13 (14.9)	
Neoadjuvant therapy				
Yes	63 (37.5)	54 (85.7)	9 (14.3)	0.223
Total resection				
Yes	145 (86.3)	115 (79.3)	30 (20.7)	0.174
No	23 (13.7)	21 (91.3)	2 (8.7)	
Siewert classification				
AEG 1	101 (60.1)	76 (75.2)	25 (24.8)	0.016
AEG 2	44 (26.2)	42 (95.5)	2 (4.5)	
AEG 3	23 (13.7)	18 (78.3)	5 (21.7)	
PD1 expression by TILs				
0 (0%)	32 (19)			
1+ (5–25%)	56 (33.3)			
2+ (26–50%)	68 (40.5)			
3+ (51–75%)	12 (7.1)			
4+ (76–100%)	0			

*AEG* adenocarcinoma of the esophagogastric junction

**Table 2:** Association of programmed cell death protein 1 (PD1) expression by tumor cells with clinicopathologic parameters in 182 patients with esophageal adenocarcinoma

Factor	Adenocarcinoma (*n* = 168) *n* (%)	PD1^+^ cancer cells (*n* = 130) *n* (%)	PD1^−^ cancer cells (*n* = 38) *n* (%)	*p* value
Tumor stage				
High-grade dysplasia	4 (2.4)	1 (25)	3 (75)	<0.001
pT1a	13 (7.7)	3 (23.1)	10 (76.9)	
pT1b	20 (11.9)	6 (30)	14 (70)	
pT2	49 (29.2)	45 (91.8)	4 (8.2)	
pT3	77 (45.8)	73 (94.8)	4 (5.2)	
pT4	5 (3)	2 (40)	3 (60)	
Lymph node status				
pNx	13 (7.7)			0.004
pN0	61 (36.3)	38 (62.3)	23 (37.7)	
pN1	31 (18.5)	27 (87.1)	4 (12.9)	
pN2	26 (15.5)	22 (84.6)	4 (15.4)	
pN3	37 (22)	33 (89.2)	4 (10.8)	
Histologic grading				
G1	7 (4.2)	5 (71.4)	2 (28.6)	0.225
G2	74 (44)	53 (71.6)	21 (28.4)	
G3	87 (51.8)	72 (82.8)	15 (17.2)	
Neoadjuvant therapy				
Yes	63 (37.5)	53 (84.1)	10 (15.9)	0.105
Total resection				
Yes	145 (86.3)	111 (76.6)	34 (23.4)	0.519
No	23 (13.7)	19 (82.6)	4 (17.4)	
Siewert classification				
AEG 1	101 (60.1)	72 (71.3)	29 (28.7)	0.058
AEG 2	44 (26.2)	39 (88.6)	5 (11.4)	
AEG 3	23 (13.7)	19 (82.6)	4 (17.4)	
PD1 expression by tumor cells				
0 (0%)	38 (22.6)			
1+ (5–25%)	36 (21.4)			
2+ (26–50%)	30 (17.9)			
3+ (51–75%)	43 (25.6)			
4+ (76–100%)	21 (12.5)			

*PD1* programmed cell death protein 1, *AEG* adenocarcinoma of the esophagogastric junction

### Expression of PD1 by TILs and by Tumor Cells

**Fig. 1:**
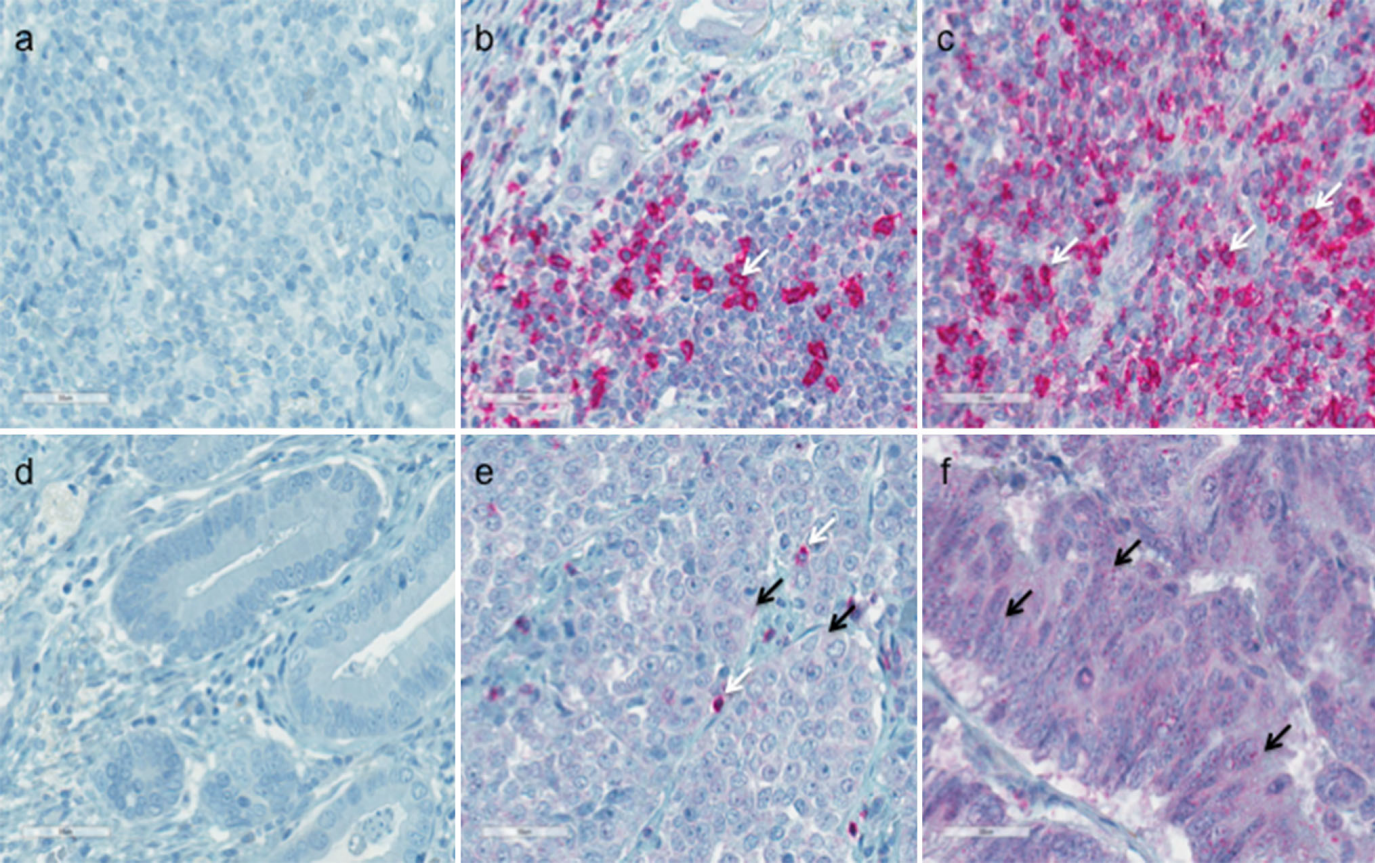
Programmed cell death protein 1 (PD1) expression on tumor-infiltrating lymphocytes (TILs) (**a**–**c**) and tumor cells (**d**–**f**) in esophageal adenocarcinoma detected by immunohistochemistry. **a** Negative PD1 staining of TILs. **b** 2+ (26–50%) positive staining of TILs. **c** 3+ (51–75%) positive staining of TILs. **d** Negative staining of tumor cells. **e** 2+ (26–50%) positive staining of tumor cells. **f** 4+ (75–100%) positive staining of tumor cells. The immunoreactivity for PD1 of tumor cells and TILs was examined at ×400 magnification, and the staining rate (percentage of tumor cells and lymphocytes showing positive staining, 0–100%) was determined. *Arrows* indicate examples for a positive PD1 staining on TILs (*white arrow*) and tumor cells (*black arrow*)

For each patient, histologic expression of PD1 was evaluated separately for TILs and tumor cells, with 136 (81%) of the 168 of patients showing high PD1 expression (>5%) on TILs (expression patterns: 0 [0%]: 19%; 1 + [5–25%]: 33%; 2 + [26–50%]: 40%; and 3 + [51–75%]: 7%) (Table 1). In 130 (77%) of the patients, PD1 expression was detected on tumor cells (expression patterns: 0 [0%]: 22.6%; 1+ [5–25%]: 21%; 2+ [26–50%]: 18%; 3+ [51–75%]: 26%; and 4+ [76–100%]: 13%) (Table 2). Figure 1 shows representative images for (a) negative (0) PD1 staining on lymphocytes, and (b) 2+ and (c) 3+ positive PD1 staining on lymphocytes as well as (d) negative (0) PD1 staining on tumor cells and (e), 2+ and (f) 4 + positive PD1 staining on tumor cells.

### Tumors Containing PD1^+^ TILs Versus Tumors Without TIL-PD1 Expression

Clinicopathologic findings were evaluated for tumors containing PD1^+^ TILs versus those without (<5%) PD1^+^ TILs (Table 1). The two groups differed significantly in terms of tumor stage (*p* < 0.001, Chi square test) and lymph node status (*p* = 0.007, Chi square test). Of the patients graded as pT3 (45.8%), 98.7% were positive for PD1 expression by TILs. In contrast, only 36.4% of the patients graded as pT1 a or b (19.6%) were positive for PD1 expression by TILs. The TILs PD1 expression patterns did not differ significantly between the patients who had received neoadjuvant therapy before surgery (*n* = 63) and those who had not received neoadjuvant therapy (*p* = 0.223; Table 1).

### Tumors Containing PD1^+^ Cancer Cells Versus Tumors Without Detectable Cancer Cell-PD1

In this study, PD1^+^ tumor cells were significantly more frequent in patients with advanced tumor stage (*p* < 0.001, Chi square test) and lymph node metastasis (*p* = 0.004) (Table 2). Almost 95% (94.8%) of the patients with stage pT3 tumors (*n* = 73) were cancer PD1^+^, compared with only 27% who had stage pT1 a and b tumors (*n* = 9). No significant difference in the frequency of PD1^+^ tumor cells could be found between the patients who received neoadjuvant therapy and those who did not (*p* = 0.105; Table 2).

### Correlation of TILs PD1^+^ and Cancer PD1^+^ Tumors With OS and DFS

In the univariate analyses, the patients with PD1^+^ (>5%) cancer cells showed a significantly lower OS than the patients in whom PD1 was not detectable on cancer cells (respective 1-, 5-, and 10-year survival rates of 74.6, 43.5, and 33.9% vs. 91.1, 68.8, and 48.1%; log-rank 0.047; Table 3). However, the PD1 status on TILs did not significantly influence OS (respective 1-, 5-, and 10-year survival rates of 75.4, 45.2, and 35.2% vs. 90, 65.9, and 45.2%; log-rank 0.132; Table 3). In terms of DFS, the cancer PD1^+^ and TIL PD1^+^ patients both demonstrated a significantly reduced DFS (5-year DFS of 71.3 vs. 41.6%; log-rank 0.008 and 73.3 vs. 41.9%; log-rank 0.008, respectively; Table 3).

**Table 3:** Uni- and multivariate Cox regression analyses estimating the influence of clinicopathologic parameters on overall survival (OS) and disease-free survival (DFS)

Factor	Univariate *p* value	Multivariate *p* value	Relative risk	95% CI
OS				
PD1 expression by TILs	0.132	0.973	1.020	0.320–3.248
PD1 expression by tumor cells	0.047	0.804	1.161	0.357–3.779
pT	0.001	0.113	1.262	0.946–1.684
pN	<0.001	<0.001	1.716	1.332–2.211
Grading	0.004	0.523	1.169	0.724–1.887
DFS				
PD1 expression by TILs	0.008	0.281	0.492	0.136–1.786
PD1 expression by tumor cells	0.008	0.348	1.820	0.521–6.352
pT	<0.001	0.022	1.524	1.063–2.185
pN	<0.001	<0.001	1.938	1.458–2.575
Grading	0.022	0.646	0.888	0.535–1.475

*CI* confidence interval, *PD1* programmed cell death protein 1, *TILs* tumor infiltration lymphocytes

### Uni- and Multivariate Analyses of the Influence of Clinicopathologic Parameters on OS and DFS

All the described clinicopathologic parameters (PD1 expression by TILs and tumor cells, pT, pN, grading, and R0 resection) showed a significant risk for both OS and DFS except for PD1 expression by TILs for OS (Table 3). However, when Cox regression analyses were performed, only lymph node status proved to be an independent risk factor for OS (hazard ratio [HR] 1.716; 95% confidence interval [CI] 1.332–2.211). The risk factors for DFS proved to be tumor status (HR 1.524; 95% CI 1.063–2.185) and lymph node status (HR 1.938; 95% CI 1.458–2.575) (Table 3).

## Discussion

This study evaluated the PD1 status of tumor cells and TILs in a large cohort of patients with esophageal adenocarcinoma. To our knowledge, this was one of the first studies aimed at comprehensively characterizing PD1 expression of this tumor entity in the TME. We detected PD1 expression on both TILs and cancer cells in esophageal tumor biospecimens, paralleling findings in human malignant melanoma.^7^,^16^,^23^ Consistent with immunohistochemical studies of normal and malignant hematopoietic and other tissues, PD1 immunoreactivity marked subsets of predominantly small TILs and larger cancer cells that exhibited both cell surface and cytoplasmic staining for PD1, with TILs showing the strongest staining intensity.^16^,^23^–^26^

Although PD1 expression often is restricted to small subsets of TILs and cancer cells in melanoma,^16^,^23^ our findings indicated that PD1^+^ cell frequencies in esophageal adenocarcinoma often exceed those in other cancers. Together, these observations provide a rationale for examining the therapeutic utility of PD1 inhibitors in patients with esophageal carcinomas, particularly those with high levels of detectable PD1 expression by TILs and cancer cells within biopsies of tumor tissue.

Importantly, we found that the presence of both PD1^+^ TILs and PD1^+^ cancer cells within the TME significantly correlated with tumor recurrence. Furthermore, high levels of cancer cell-expressed PD1 within patients’ tumor biopsies significantly correlated with decreased OS in our patient cohort. Although these differences were not independent in multivariate analyses, these findings identified PD1 as a potential biomarker of tumor virulence in esophageal carcinoma. Moreover, they support the possibility that the PD1 pathway might also functionally promote tumor virulence in esophageal carcinoma given its elevated expression in late tumor stages and correlation with adverse patient outcome.

Our results further suggest that esophageal carcinoma, like melanoma, could exploit the PD1 pathway to promote cancer progression both by dampening tumor-specific immunity via engagement of TIL-expressed PD1 and by triggering tumor cell-intrinsic growth signals via engagement of cancer cell-expressed PD1. However, whether PD1 does indeed modulate the antitumor immune response and/or function as a tumor cell-intrinsic growth receptor in this malignancy requires future dedicated studies. In a separate study,^13^ PD1 and PD-L1 expression were assessed previously in tumor biospecimens from 349 patients with esophageal squamous cell carcinoma, and PD-L1 expression levels were found to correlate significantly with favorable outcome, whereas PD1 expression within the TME did not show any significant associations with clinicopathologic parameters.^13^

The pathophysiology of esophageal squamous cell carcinoma differs from that of esophageal adenocarcinoma. The latter is marked by a high somatic mutation rate,^27^ presumably because of frequent exposure to gastric fluids and subsequent chronic inflammation. Because both a high mutational burden and chronic inflammation have been linked to PD1 receptor expression in patients with chronic inflammatory liver diseases 28 as well as in patients with other cancers, including non-small cell lung cancer (NSCLC),^29^ we were especially interested in PD1 expression patterns in patients with esophageal adenocarcinoma.

To date, only one study has analyzed PD1 pathway member expression in esophageal adenocarcinoma, with an emphasis on assessing expression of the PD1 ligands PD-L1 and PD-L2. This study, conducted by Derks et al.,^30^ investigated tissue microarrays containing esophageal tumor tissue cores as well as benign tissue controls obtained from 354 patients. The authors detected PD-L2 expression in tissues associated with Barrett’s esophagus and reported a potential association of the inflammatory environment in Barrett’s esophagus and PD1 ligand expression.^30^ A potential limitation of this study was the use of tissue microarrays instead of whole tissue slides, especially because esophageal adenocarcinomas are highly heterogeneous.^31^,^32^ Consequently, several important areas of the tumor might have been missed with analyses limited to microarray-based tissue cores. Furthermore, PD1 expression by cancer cells was not evaluated in the study by Derks et al.,^30^ and areas lacking a T-cell infiltrate often were excluded from the analysis.^13^

In our study, patients with esophageal adenocarcinoma expressed PD1 on both TILs and tumor cells. Importantly, these expression patterns correlated with the patients’ tumor stage and outcome. Several phase 3 trials demonstrated improved OS for melanoma, NSCLC, and renal cell carcinoma patients treated with PD1-blocking antibodies,^33^–^38^ resulting in Food and Drug Administration (FDA) approval of two PD1 inhibitors as second-line therapies for patients with these malignancies.

Regarding gastroesophageal cancer, multiple studies currently are focused on blocking the PD1 pathway.^39^–^42^ One of these studies is the recently started phase 3 clinical trial with nivolumab treatment for patients with unresectable advanced or recurrent gastric cancer, including gastroesophageal junction cancer (ONO-4538-12).

In this study, we found that in our cohort of 168 esophageal adenocarcinoma patients, 81% (*n* = 136) showed PD1 expression by TILs and 77.4% (*n* = 130) demonstrated PD1 expression by tumor cells. Given the broad success of PD1 pathway blockade, our findings of PD1 expression in esophageal carcinoma provide a strong rationale for evaluating the therapeutic utility of PD1 inhibitors in this group of patients. Clinical trials are warranted in this regard. Besides diminishing the protective effect of PD1 ligand expression on receptor activation on immune cells and thus abrogating the antitumoral response, tumor growth might also be reduced via direct inhibition of PD1 on esophageal adenocarcinoma tumor cells themselves.
